# Associations of frailty with the incidence and progression trajectory of cardiometabolic-kidney multimorbidity: insights from multi-state modelling

**DOI:** 10.7189/jogh.16.04133

**Published:** 2026-04-30

**Authors:** Minghui Han, Jing Zhou, Wenxin Bai, Zhenzhen Wan, Meng Chen, Mengli Yan, Wei Feng, Ge Wang, Jing Zhang, Lina Zhang, Lei Yan, Fengmin Shao, Yue Gu

**Affiliations:** 1 Henan Provincial Clinical Research Centre for Kidney Disease, Henan Provincial Key Laboratory of Kidney Disease and Immunology, Department of Nephrology, Henan Provincial People’s Hospital, Zhengzhou University People’s Hospital, Zhengzhou, Henan, China; 2 Department of Nephrology, Fuwai Central China Cardiovascular Hospital, Zhengzhou, Henan, China

## Abstract

**Background:**

The American Heart Association recently introduced the cardiovascular-kidney-metabolic concept, but the impacts of frailty status on the disease trajectories remain unknown. We aimed to investigate the role of frailty status in the trajectories from being free of cardiometabolic-kidney disease (CMKD) to first CMKD (FCMKD), then to cardiometabolic-kidney multimorbidity (CMKM), and finally to death.

**Methods:**

In this prospective cohort study, we included 392 902 participants aged 37–73 years from the UK Biobank. We assessed frailty using the Fried criteria’s frailty phenotype, based on five individual components. We used multistate models to estimate hazard ratios (HRs) and 95% confidence intervals (CIs).

**Results:**

Among 392 902 participants, 53 845 developed FCMKD, 8025 developed CMKM, and 25 538 died during a median follow-up of 13.49 years. Frailty was associated with a higher risk of transition from healthy to FCMKD (HR = 2.08; 95% CI = 1.99–2.17) than from FCMKD to CMKM (HR = 1.47; 95% CI = 1.34–1.62) (*P* < 0.001). Frailty showed a stronger association for healthy to death than that of transitions from FCMKD or CMKM to death (HR = 2.52; 95% CI = 2.34–2.72, *P* < 0.001). When splitting FCMKD into four CMKDs, the risks of disease-specific transitions associated with frailty varied, with the higher risk for chronic kidney disease to CMKM observed than that for other CMKDs to CMKM (HR = 2.11; 95% CI = 1.66–2.68). Consistent associations were also observed for pre-frailty.

**Conclusions:**

Both frailty and pre-frailty played key but different roles in disease transitions from healthy to FCMKD, to CMKM, and further to death, and had diverse impacts on disease-specific transitions of CMKDs. Our findings underscore the significance of early detection and interventions for frailty to prompt the comprehensive care of CMKM.

In 2023, the American Heart Association introduced the concept of the cardiovascular-kidney-metabolic syndrome, attributable to the complex interplay among obesity, diabetes, chronic kidney disease (CKD), and cardiovascular disease (CVD) [1]. Rather than simply considering cardiometabolic disease, typically including diabetes, stroke, and coronary heart disease (CHD), and CKD separately as previously, this framework advocates for integrated screening, prevention, and management of cardiometabolic-kidney disease (CMKD) because of their shared risk factors and common therapeutic strategies [2–5]. Identifying potential risk factors and assessing the impacts of CMKD on disease transitions would be highly significant in alleviating the health burden.

Frailty, characterised by decreased reserves across multiple systems and increased vulnerability to stressors, has attracted greater attention due to an ageing population [6,7]. Recent studies have reported that frailty was positively associated with increased risks of diabetes, CKD, and CVD [8–11]. Prior studies also revealed that frailty conferred high risks of CVD among patients with diabetes or CKD [12,13]. Generally, these studies either explored the impact of frailty on the incidence of single CMKDs in participants without any CMKD, regardless of potential disease transitions [9–11], or on the prognosis of patients with a single CMKD [12,13]. Although the detrimental role of frailty was identified in these studies, the fragmented analyses made it challenging to compare effects across disease stages before and after a single CMKD. Evaluating and comparing the impact of frailty on the incidence and progression of cardiometabolic-kidney multimorbidity (CMKM) simultaneously would allow us to understand the holistic influence of frailty. This can promote primary and secondary prevention of CMKM within the cardiovascular-kidney-metabolic health framework [3,14].

Therefore, we aimed to investigate the potentially different effects of frailty and its components on trajectories from being free of CMKD to first CMKD (FCMKD), then to CMKM, and finally to death. Given the large number of cases, we further split FCMKDs into diabetes, CKD, stroke, and CHD and explored associations across transition paths by CMKD.

**Adherence to JoGH’s Guidelines for Reporting Analyses of Big Data Repositories Open to the Public (GRABDROP)**

We followed the Journal of Global Health Guidelines for reporting secondary analyses of publicly available large international data repositories (GRABDROP) [15]. Detailed adherence to each GRABDROP item is provided in Checklist S1 in the **Online Supplementary Document**).

## METHODS

### Study design and participants

UK Biobank is a prospective, population-based cohort study that recruited more than 500 000 participants aged 37–73 years between 2006 and 2010 [16]. Participants attended one of 22 assessment centres across England, Scotland, and Wales, completed a touchscreen questionnaire and nurse-led interview, had physical measurements, and provided biological samples at baseline. This study was approved by the North West Multi-Centre Research Ethics Committee (11/NW/0382), and all participants gave written informed consent.

Of 502 366 participants in the UK Biobank, 81 559 participants with diabetes, CKD, stroke, and CHD were excluded. After excluding 27 905 participants with missing data on five frailty indicators (weight loss, exhaustion, physical activity, walking speed, and grip strength), we included a total of 392 902 participants without CMKD in the final analyses (Figure S1 in the **Online Supplementary Document**).

### Outcome ascertainment

We obtained data on primary care, hospital inpatient, and death registers to ascertain the outcomes. All disease events were defined according to the International Classification of Diseases (10th revision): CHD (I20–25), stroke (I60, I61, I63, and I64), CKD (N18), and diabetes (E11). For baseline disease status, we additionally defined CKD as estimated glomerular filtration rate <60 mL/min/1.73 m^2^, and/or urinary albumin to creatinine ratio ≥30 mg/g [17]. Diabetes was additionally diagnosed based on random glucose ≥11.1 mmol/L, glycated haemoglobin ≥48 mmol/L (6.5%), or insulin use. The time-to-event endpoint was calculated from the date of baseline enrolment until the earliest of the date of outcome occurrence, the date of loss to follow-up, and the last date of follow-up (31 October 2022 for England, 31 May 2022 for Wales, and 31 August 2022 for Scotland). CMKD was defined as having one of four diseases, including diabetes, CKD, stroke, and CHD. CMKM was defined as the coexistence of two or more CMKD diseases.

### Frailty assessment

We assessed frailty status using the five frailty indicators (weight loss, exhaustion, physical activity, walking pace, and grip strength) originally described by Fried and colleagues [18]. We adapted some of these items to fit the data available in the UK Biobank, as in other published studies [8,19,20]. Participants answering ‘yes – lost weight’ were categorised as weight loss to the question ‘Compared with one year ago, has your weight changed?’ and those answering ‘no – weight about the same’ or ‘yes – gained weight’ were categorised as not. Regarding the question for exhaustion, ‘Over the past two weeks, how often have you felt tired or had little energy?’ participants answering ‘more than half the days’ or ‘nearly every day’ were grouped into exhaustion, and those answering ‘several days’ or ‘not at all’ were considered not. Physical activity was derived from the activity types and frequency in the past four weeks, and low physical activity was defined when answering ‘no or light activity with a frequency of once per week or less.’ Walking pace was classified as slow or normal (steady or brisk) based on self-reported data. Grip strength of two hands was measured using the Jamar J00105 hydraulic hand dynamometer, and we used the higher value in this study. Low grip strength was defined using sex and body mass index (BMI) adjusted cutoffs (Table S1 in the **Online Supplementary Document**). We classified participants as frail (meeting three or more criteria), pre-frail (meeting one or two criteria), or non-frail.

### Covariates

We included age, sex, assessment centres, ethnicity (White or other), education (degree level/professional education or other), Townsend deprivation index, smoking status (current smoking or not), drinking status (drinking ≥3 times a week or not), healthy diet (yes or not), family history of cardiometabolic disease, obesity, hypertension (yes or not), lipid-lowering drugs use (yes or not), and elevated low-density lipoprotein cholesterol (LDL-C) as potential confounders. We defined a healthy diet as meeting at least five items of the recommendations (adequate consumption: fruit, vegetables, whole grains, fish, shellfish, dairy products, and vegetable oils; reduced consumption: refined grains, processed meats, unprocessed meats, and sugar-sweetened beverages) [21]. Participants whose first-degree relatives (including father, mother, and siblings) have diabetes, stroke, or CHD were defined as having a family history of cardiometabolic disease. Obesity was defined as BMI ≥ 30 kg/m^2^. Hypertension was defined as self-reported, and/or systolic blood pressure ≥140 mm Hg, and/or diastolic blood pressure ≥90 mm Hg, and/or using antihypertensive medication. Elevated LDL-C was defined as LDL-C ≥ 3.0 mmol/L.

### Statistical analyses

We presented baseline characteristics by frailty status (non-frail, pre-frail, and frail) as means and standard deviations (SDs) for continuous variables or numbers (percentages) for categorical variables. We used a generalised linear model or a Cochran-Armitage trend test to compare differences. Differences between included and excluded participants were compared using *t* tests and the χ^2^ test.

**Figure 1:**
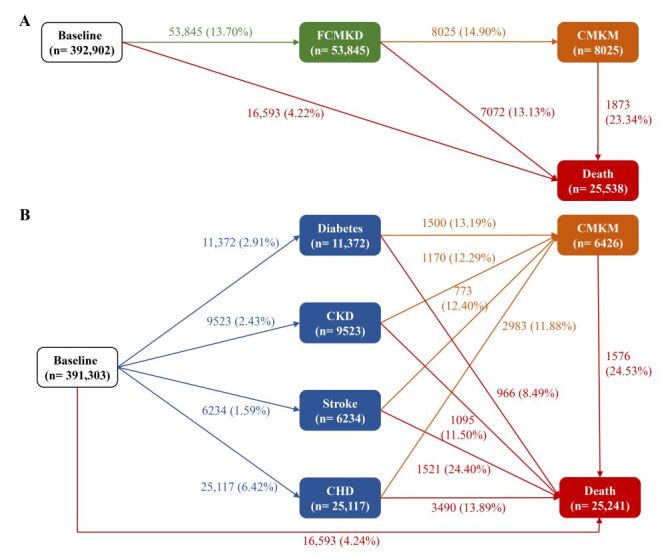
Numbers (percentages) of participants for disease transitions. **Panel A.** Transition pattern A from baseline to first cardiometabolic-kidney disease (FCMKD), and then to cardiometabolic-kidney multimorbidity (CMKM), and subsequently to death. CMKD was defined as having one of four diseases, including diabetes, CKD, stroke, and CHD. CMKM was defined as the coexistence of two or more CMKD diseases. **Panel B.** Transition pattern B from baseline to one of four CMKDs (diabetes, chronic kidney disease, stroke, and coronary heart disease), CMKM, and death.

We used a multi-state model to assess the role of both frailty status and frailty score (continuous variable) in the temporal progression of disease from being free of CMKD to FCMKD, CMKM, and death. The multi-state model is an extension of the competing risk model and offers a unique advantage in exploring how certain factors influence different stages of disease progression simultaneously [22]. We constructed five transition stages (transition pattern A) (**Figure 1**, Panel A): baseline healthy to FCMKD, FCMKD to CMKM, baseline healthy to death, FCMKD to death, and CMKM to death. We tested the transition-specific proportional hazards assumptions using Schoenfeld residuals and stratified covariates that violated them, thereby satisfying the assumption. For participants entering different stages on the same date, we calculated the entry date of the theoretically prior state as the entry date of the later state minus 0.5 days. For example, for participants who died of FCMKD, the date of FCMKD incidence equals the date of death minus 0.5 days. Additionally, the independent associations between the five frailty indicators and risks of disease transitions were also investigated. We used the function ‘heterogeneity’ in *R*, version 4.4.2 (R Core Team, Vienna, Austria) to calculate the *P*-value for heterogeneity. Significant heterogeneity indicates differential risk across associations of frailty and its components with disease transitions (Methods S1 in the **Online Supplementary Document**) [23]. We estimated the transition probabilities at one, two, five, 10, and 15 years for each disease transition, with covariates being set to the average levels of the study population.

To further evaluate the effects of frailty on disease progression by individual CMKDs, we split FCMKDs into four individual diseases (*i.e.* diabetes, CKD, stroke, and CHD), resulting in 14 transition stages (transition pattern B) (**Figure 1**, Panel B). Participants who were diagnosed with at least two of FCMKDs on the same date were excluded from this analysis (n = 1599) because we could not ascertain the temporal sequence of FCMKDs.

We further conducted subgroup analyses by age (<60 *vs*. ≥60 years) and sex to examine potential effect modification. We conducted several sensitivity analyses to assess the robustness of our results (Methods S2 in the **Online Supplementary Document**). We performed the multi-state model using ‘mstate’ package in *R*. We considered a two-sided *P*-value <0.05 statistically significant.

## RESULTS

### Descriptive analysis

Of the 392 902 included participants, the mean age was 56.33 years (SD = 8.06), and 43.49% were men (**Table 1**). In this study, 141 014 (35.89%) participants were considered pre-frail, and 9906 (2.52%) were considered frail. During a median follow-up time of 13.49 years (**Figure 1**, Panel A), 53 845 (13.70%) participants experienced at least one FCMKD. Of all incident FCMKD cases, 8025 (14.90%) participants developed CMKM, 1873 (23.34%) died from any cause afterwards, and 7072 (13.13%) died without developing CMKM. When FCMKDs were further divided into four individual diseases, 11 372 (2.91%) participants developed diabetes, 9523 (2.43%) developed CKD, 6234 (1.59%) developed stroke, 25 117 (6.42%) developed CHD, and more than 10% of each disease group suffered CMKM afterwards (**Figure 1**, Panel B; Table S2 in the **Online Supplementary Document**).

**Table 1:** Baseline characteristics of study participants*

Characteristics	Overall (n = 392 902)	Non-frailty (n = 241 982)	Pre-frailty (n = 141 014)	Frailty (n = 9906)	*P*-value
Age in years, x̄ (SD)	56.33 (8.06)	56.26 (8.03)	56.42 (8.13)	57.09 (7.79)	<0.001
Men	170 886 (43.49)	112 022 (46.29)	55 886 (39.63)	2978 (30.06)	<0.001
White	374 538 (95.61)	233 796 (96.88)	131 951 (93.88)	8791 (89.12)	<0.001
Townsend index, x̄ (SD)	–1.47 (2.99)	–1.75 (2.82)	–1.12 (3.14)	0.26 (3.53)	<0.001
Higher education	194 207 (49.82)	128 251 (53.34)	63 006 (45.13)	2950 (30.30)	<0.001
Current smoker	39 381 (10.05)	20 972 (8.69)	16 482 (11.73)	1927 (19.59)	<0.001
Drinking ≥3 times a week	178 145 (45.37)	120 727 (49.91)	55 175 (39.16)	2243 (22.70)	<0.001
Healthy diet	48 324 (12.30)	30 238 (12.50)	17 057 (12.10)	1029 (10.39)	<0.001
Family history of CMD	226 478 (57.84)	136 898 (56.73)	83 332 (59.35)	6248 (63.55)	<0.001
Obesity	82 955 (21.11)	38 859 (16.06)	39 443 (27.97)	4653 (46.97)	<0.001
Hypertension	193 293 (49.20)	115 830 (47.87)	71 680 (50.83)	5783 (58.38)	<0.001
Lipid-lowering drugs use	38 301 (9.80)	21 117 (8.77)	15 620 (11.15)	1564 (16.08)	<0.001
Elevated LDL-C	288 070 (77.76)	179 186 (78.35)	102 042 (76.95)	6842 (74.82)	<0.001

CMD – cardiometabolic disease, LDL-C – low-density lipoprotein cholesterol, SD – standard deviation, x̄ – mean

*Presented as n (%) unless specified otherwise.

### Multi-state analyses for frailty

Multistate analyses showed that frailty was associated with increased risks of all disease transitions in the multivariable-adjusted model, with different magnitudes of association observed (**Table 2**) (*P* < 0.001). There was a stronger association for transition from baseline healthy to FCMKD than from FCMKD to CMKM (*P* < 0.001), with hazard ratios (HRs) of 2.08 (95% confidence interval (CI) = 1.99–2.17) and 1.47 (95% CI = 1.34–1.62), respectively. Frailty conferred a higher risk of transition from baseline health to death (HR = 2.52; 95% CI = 2.34–2.72) than transitions from FCMKD (HR = 1.49; 95% CI = 1.33–1.67) or CMKM (HR = 1.28; 95% CI = 1.06–1.54) to death (*P* < 0.001). Similar associations for pre-frailty were observed, and the adjusted HRs were 1.30 (95% CI = 1.28–1.33) for baseline healthy to FCMKD, 1.14 (95% CI = 1.09–1.20) for FCMKD to CMKM, 1.30 (95% CI = 1.25–1.34) for baseline healthy to death, 1.19 (95% CI = 1.13–1.25) for FCMKD to death, and 1.11 (95% CI = 1.00–1.23) for CMKM to death. Participants with frailty showed increased probabilities of progressing to FCMKD, subsequently to CMKM, and ultimately to death (Table S3 in the **Online Supplementary Document**).

**Table 2:** Hazard ratios (95% CIs) for transition pattern A associated with frailty*

Items	Pre-frailty		Frailty		Per one-indicator
	**Cases, n**	**HR (95% CI)**	**Cases, n**	**HR (95% CI)**	**HR (95% CI)**
Baseline to FCMKD	22 868	1.30 (1.28–1.33)	2702	2.08 (1.99–2.17)	1.25 (1.24–1.27)
FCMKD to CMKM	3679	1.14 (1.09–1.20)	589	1.47 (1.34–1.62)	1.13 (1.11–1.16)
Baseline to death	6667	1.30 (1.25–1.34)	891	2.52 (2.34–2.72)	1.29 (1.27–1.32)
FCMKD to death	3149	1.19 (1.13–1.25)	441	1.49 (1.33–1.67)	1.13 (1.10–1.17)
CMKM to death	872	1.11 (1.00–1.23)	161	1.28 (1.06–1.54)	1.09 (1.04–1.14)

CI – confidence interval, CMKM – cardiometabolic-kidney multimorbidity, FCMKD – first cardiometabolic-kidney disease, HR – hazard ratio

*Adjusted for age, sex, ethnicity, region, education levels, Townsend deprivation index, smoking status, drinking status, healthy diet, family history of cardiometabolic disease, obesity, hypertension, lipid-lowering drugs use, and elevated low-density lipoprotein cholesterol.

**Figure 2:**
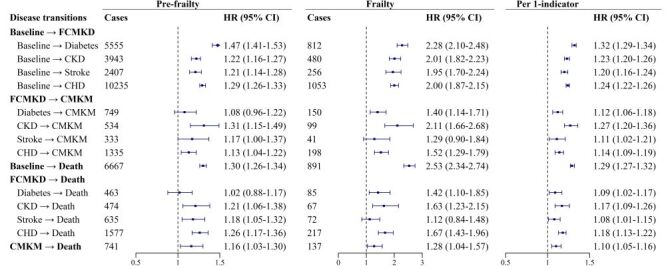
Hazard ratios (95% CIs) for transition pattern B associated with frailty. Adjusted for age, sex, ethnicity, region, education levels, Townsend deprivation index, smoking status, drinking status, healthy diet, family history of cardiometabolic disease, obesity, hypertension, lipid-lowering drugs use, and elevated low-density lipoprotein cholesterol. CHD – coronary heart disease, CKD – chronic kidney disease, CMKM – cardiometabolic-kidney multimorbidity, FCMKD – first cardiometabolic-kidney disease, HR – hazard ratio.

After splitting FCMKDs into four specific CMKDs, we found that frailty was associated with increased risks of transitions from baseline healthy to CMKDs, with similar magnitudes (**Figure 2**). However, for CMKDs to CMKM, the association for CKD (HR = 2.11; 95% CI = 1.66–2.68) was stronger than that for diabetes (HR = 1.40; 95% CI = 1.14–1.71), stroke (HR = 1.29; 95% CI = 0.90–1.84), and CHD (HR = 1.52; 95% CI = 1.29–1.79) (*P* < 0.05). Besides, pre-frailty had a larger effect on transition from baseline healthy to diabetes (HR = 1.47; 95% CI = 1.41–1.53) than that for CKD (HR = 1.22; 95% CI = 1.16–1.27), stroke (HR = 1.21; 95% CI = 1.14–1.28), and CHD (HR = 1.29; 95% CI = 1.26–1.33) (*P* < 0.001). There was also a stronger association of pre-frailty for transition from CKD to CMKM than that for diabetes to CMKM (*P* < 0.05), a marginally stronger association for CHD to CMKM (*P* = 0.051), and a similar association for stroke to CMKM (*P* = 0.230). For CMKDs to death, frailty tended to have a greater impact on transition for CHD or CKD than that for the other two CMKDs, but the differences were non-significant (*P* > 0.05).

### Multi-state analyses for frailty indicators

For transition pattern A, a slow walking pace conferred the highest risk for baseline healthy to FCMKD (HR = 1.47; 95% CI = 1.43–1.52), followed by exhaustion (HR = 1.28; 95% CI = 1.25–1.32). Their estimates were larger than those for the remaining three frailty indicators (*P* < 0.01) (**Table 3**). For FCMKD to CMKM and baseline healthy to death, slow walking pace showed the strongest associations, with HRs of 1.30 (95% CI = 1.21–1.39) and 1.81 (95% CI = 1.72–1.92), respectively. Low physical activity, slow walking pace, and low grip strength were positively associated with 18%, 32%, and 20% higher risks of FCMKD to death, respectively, but were not associated with weight loss or exhaustion. A slow walking pace was also associated with an elevated risk of CMKM to death (HR = 1.31; 95% CI = 1.14–1.50), whereas not for the other four frailty indicators.

**Table 3:** Hazard ratios (95% CIs) by frailty indicators in transition pattern A*

Items	Weight loss, HR (95% CI)	Exhaustion, HR (95% CI)	Low physical activity, HR (95% CI)	Slow walking pace, HR (95% CI)	Low grip strength, HR (95% CI)
Baseline to FCMKD	1.13 (1.10–1.16)	1.28 (1.25–1.32)	1.19 (1.16–1.23)	1.47 (1.43–1.52)	1.22 (1.19–1.25)
FCMKD to CMKM	1.08 (1.02–1.15)	1.09 (1.02–1.16)	1.07 (1.00–1.15)	1.30 (1.21–1.39)	1.12 (1.06–1.19)
Baseline to death	1.17 (1.12–1.22)	1.16 (1.10–1.22)	1.22 (1.16–1.29)	1.81 (1.72–1.92)	1.22 (1.16–1.27)
FCMKD to death	1.02 (0.95–1.09)	0.95 (0.89–1.03)	1.18 (1.09–1.27)	1.32 (1.22–1.42)	1.20 (1.12–1.27)
CMKM to death	1.05 (0.93–1.20)	0.97 (0.85–1.11)	1.02 (0.88–1.18)	1.31 (1.14–1.50)	1.08 (0.95–1.21)

CI – confidence interval, CMKM – cardiometabolic-kidney multimorbidity, FCMKD – first cardiometabolic-kidney disease, HR – hazard ratio

*Adjusted for age, sex, ethnicity, region, education levels, Townsend deprivation index, smoking status, drinking status, healthy diet, family history of cardiometabolic disease, obesity, hypertension, lipid-lowering drugs use, and elevated low-density lipoprotein cholesterol. For analyses of dichotomous frailty indicators, five frailty indicators were mutually adjusted.

**Figure 3:**
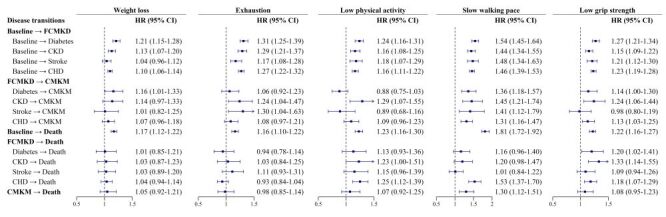
Associations between frailty indicators and risks of disease transitions in transition pattern B. Adjusted for age, sex, ethnicity, region, education levels, Townsend deprivation index, smoking status, drinking status, healthy diet, family history of cardiometabolic disease, obesity, hypertension, lipid-lowering drugs use, and elevated low-density lipoprotein cholesterol. For analyses of dichotomous frailty indicators, five frailty indicators were mutually adjusted. CHD – coronary heart disease, CKD – chronic kidney disease, CMKM – cardiometabolic-kidney multimorbidity, FCMKD – first cardiometabolic-kidney disease, HR – hazard ratio.

For transition pattern B, all five frailty indicators were positively associated with increased risks for the transition from baseline healthy to FCMKD, except for weight loss in the transition from baseline healthy to stroke (**Figure 3**). Slow walking pace conferred the highest risk for baseline healthy-to-FCMKD (*P* < 0.05). For specific CMKDs to CMKM, a slow walking pace was positively associated with increased risks for all transitions, and low grip strength was also associated with increased risks, except for the stroke transition. Additionally, positive associations were also observed for weight loss in the transition for diabetes, for exhaustion in the transition for CKD and stroke, and for low physical activity in the transition for CKD. For FCMKD to death, low physical activity and low grip strength were positively associated with increased risks for CKD or CHD to death. In addition, positive associations were observed for slow walking pace during the transition for CHD and for low grip strength during the transition for diabetes.

### Subgroup and sensitivity analyses

Pre-frailty and frailty showed similar associations with disease transitions (except for CMKM to death) across sub-populations by sex and age (Table S4–7 in the **Online Supplementary Document**). Frailty was positively associated with increased risk for CMKM to death among men and older people, while not for women or young people. In sensitivity analyses, the associations of both pre-frailty and frailty with disease transitions were consistent with those in the main analyses (Table S8 and S9 in the **Online Supplementary Document**).

## DISCUSSION

Using a large-scale prospective cohort, we systematically assessed the role of frailty across all disease transition stages from healthy to FCMKD, to CMKM, and to death. We found positive associations between frailty status and all disease transition stages, with varying magnitudes. Frailty and pre-frailty showed stronger associations with the transition from healthy to FCMKD than with the transition from FCMKD to CMKM, and the strongest association for the healthy-to-death transition among the mortality outcomes. When FCMKD was divided into four specific CMKDs, frailty had a similar impact on transitions from healthy to each CMKD, while it conferred the highest risk of CKD to CMKM among transitions from CMKDs to CMKM. For pre-frailty, different associations were observed among transitions from healthy to four CMKDs and from CMKDs to CMKM. When examining the role of frailty indicators, we found that five indicators played an important role in transitions, but to varying degrees.

Several concepts, such as cardiometabolic disease, cardiorenal syndrome, and metabolic syndrome, have been constructed to promote the integrated management of cardiovascular, metabolic, and kidney diseases [24–26]. Given the fact that these terms were considered as different entities, cardiovascular-kidney-metabolic syndrome was defined based on their intricate overlapping relations [1]. The present study included diabetes, CKD, stroke, and CHD as outcomes due to their poor prognosis and multidirectional relationships according to the cardiovascular-kidney-metabolic framework. We timely evaluate the effects of frailty on the development, progression, and prognosis of CMKM, which provides important insights and implications for managing cardiovascular-kidney-metabolic syndrome. The American Heart Association also points out that the competing risks should be considered when estimating the risks of these diseases [3]. Different from the traditional Cox models, the multi-state model could consider all outcomes during disease transitions of CMKM simultaneously, and rule out the competing risks [27].

Unlike prior studies that focused on a single disease stage, our study examined the adverse effects of frailty across all disease transitions in CMKM using a multi-state model. Several studies reporting positive associations between frailty and single CMKDs were consistent with our results regarding the transition from healthy to FCMKD [9–11]. Two prospective studies conducted in Chinese and American populations separately revealed detrimental impacts of frailty on CVD among patients with diabetes and CKD [12,13], which were similar to our findings for FCMKD to CMKM. Due to differences in study populations, analytic strategies, and potential competing risks, it is not appropriate to infer the role of frailty in transitions from healthy to FCMKD, to CMKM, and then to death across these studies. The present study found that frailty conferred a higher risk of the transition from healthy to FCMKD than from FCMKD to CMKM, and a greater risk of the transition from healthy to death than from FCMKD or CMKM to death. These findings not only demonstrated the different roles of frailty in both primary and secondary prevention of CMKM but also indicated greater benefits achieved if reversing frailty to non-frailty before developing FCMKD [14,28]. Moreover, we observed similar associations between participants aged <60 and ≥60 years, except for the transition from CMKM to death. The findings indicate that we should not focus solely on the consequences of frailty in the elderly, and that intervention as early as possible is necessary whenever frailty is present [8,29].

The large sample size and long follow-up period allowed us to detail the transitions of four CMKDs and facilitated direct comparisons of estimates of frailty status effects. Despite the similar magnitude of associations from healthy to CMKDs, we found that frailty had the greatest impact on the transition from CKD to CMKM among CMKD transitions. The findings demonstrate the plausibility of cardiovascular-kidney-metabolic syndrome that incorporates CKD and cardiometabolic disease into holistic care [30,31]. Notably, pre-frailty conferred the largest risk for healthy to diabetes among the transitions for healthy to CMKDs, and relatively larger risk for CKD or stroke to CMKM among the transitions for CMKDs to CMKM. These findings for pre-frailty also had significant public health implications due to their large proportion. More than 35% participants in the current study were considered pre-frail, and identifying those specific sub-populations early and performing timely intervention would help reduce the burden of CMKM among the middle-aged and older adults [6,28,32,33]. Our findings suggest integrating frailty assessment and intervention into routine cardiovascular-kidney-metabolic care, such as incorporating mobility assessments as a routine vital sign, implementing functional reserve screening for high-risk groups, especially those with frailty or pre-frailty, and developing early frailty-reversal programmes for individuals with CMKDs.

Our study also found that frailty indicators, especially slow walking pace, play important roles in the transitions of CMKM. Similarly, previous studies found positive associations between slow walking pace and increased risks of diabetes, CKD, and CVD [34–36]. Given the emphasis on walking step counts [37], increasing walking speed may also help prevent and manage CMKM. Nevertheless, we failed to observe a positive association between weight loss and exhaustion and transitions from FCMKD or CMKM to death, which may be due to their small effects and limited incident events.

### Strengths and limitations

The main strengths of our study are the use of the multi-state model and the selection of CMKM as the outcomes under the concept of cardiovascular-kidney-metabolic syndrome. The former could enable us to explore and compare the impacts of frailty across different stages of CMKM while accounting for potential competing risks, and the latter could provide vital insights into understanding and managing cardiovascular-kidney-metabolic syndrome. Additionally, the large sample size and adequate incident events allowed us to further investigate all disease transitions of individual CMKDs with substantial statistical power. Our study also had several limitations. First, frailty was assessed using a combination of self-reported characteristics and objective measurements, which may lead to misclassification. However, a prior study has shown that the characteristics are similar when assessing frailty with self-report and test-based measures [38], and several studies have validated such a frailty assessment [8,20]. Second, we used baseline frailty status and did not account for potential changes during follow-up. However, limited data are available from the UK Biobank on how frailty change influences disease progression in CMKM. Third, the exclusion of participants with missing frailty data may have introduced selection bias, as these individuals were more likely to have socioeconomic disadvantages. Fourth, deviations from the Markov assumption were observed in certain transitions. However, our primary results focus on transition-specific HRs, whereas the violation primarily affects the accuracy of long-term cumulative transition probabilities rather than the transition-specific relative effects. Finally, because our study was conducted in the UK population, which is predominantly White, further studies in other populations or ethnicities are needed to validate our findings.

## CONCLUSIONS

In this large population-based cohort study, we found that both frailty and pre-frailty were positively associated with disease transitions from healthy to FCMKD, to CMKM, and then to death, with varying degrees. Additionally, frailty status also showed diverse associations with disease-specific transitions by CMKDs. Our findings highlight the need to closely monitor and manage frailty to reduce the disease burden of CMKM. It would be highly significant to incorporate these findings into disease management pathways within the cardiovascular-kidney-metabolic health framework.

## Additional material


Online Supplementary Document
