# Supplementary material for: Associations of frailty with the incidence and progression trajectory of cardiometabolic-kidney multimorbidity: insights from multi-state modelling
Source: J Glob Health. 2026 Apr 30;16:04133. doi: 10.7189/jogh.16.04133 (PMC13129893; doi:10.7189/jogh.16.04133)
Supplement: Online Supplementary Document [file jogh-16-04133-s001.pdf]

Supplement to: Han M, Zhou J, Bai W, Wan Z, Chen M, Yan M, Feng W, Wang G, Zhang J, Zhang L, Yan L, Shao F, Gu Y. Associations of frailty with the incidence and progression trajectory of cardiometabolic-kidney multimorbidity: insights from multi-state modelling. J Glob Health. 2026;16:04133.

List of contents

Supplementary methods. Heterogeneity test

Supplementary methods. Sensitivity analysis

Table S1. Outline of JoGH's Guidelines for Reporting Analyses of Big Data Repositories Open to the Public (GRABDROP) items

Table S2. Frailty criteria.

Table S3. Baseline characteristics of included and excluded participants.

Table S4. Transition probabilities over years for transition pattern A according to frailty status.

Table S5. Hazard ratios (95% CIs) for transition pattern A associated with frailty stratified by sex.

Table S6. Hazard ratios (95% CIs) for transition pattern A associated with frailty stratified by age.

Table S7. Hazard ratios (95% CIs) for transition pattern B associated with frailty stratified by sex.

Table S8. Hazard ratios (95% CIs) for transition pattern B associated with frailty stratified by age.

Table S9. Sensitivity analyses for transition pattern A.

Table S10. Sensitivity analyses for transition pattern B.

Figure S1. Flow chart of study participants included and excluded in the analyses.

STROBE statement

Supplemental methods. Heterogeneity test

The R function "heterogeneity" was used to calculate *P* value for heterogeneity [1].

```
heterogeneity <- function(beta, se) {
    # beta must be a vector of estimates
    # se must be a vector of their standard errors
    # df denotes degrees of freedom
    df <- length(beta) - 1
    # expected_beta: inverse variance weighted average of betas
    expected_beta <- sum(beta / se^2) / sum(1 / se^2)
    heterogeneity_test_statistic <- sum(((beta - expected_beta) / se)^2)
    p <- pchisq(heterogeneity_test_statistic, df = df, lower = FALSE)
    return(list("test statistic" = heterogeneity_test_statistic, "p" = p))
    }
# example: p-value for heterogeneity by sex (beta coefficient and SE in men: beta1, se1; women: beta2, se2)
heterogeneity(beta=c(beta1, beta2), se=c(se1, se2))
```

Reference

1. Pang Y, Han Y, Yu C, Kartsonaki C, Guo Y, Chen Y, Yang L, Du H, Hou W, Schmidt D *et al.* **The role of lifestyle factors on comorbidity of chronic liver disease and cardiometabolic disease in Chinese population: A prospective cohort study**. *Lancet Reg Health West Pac* 2022, **28**:100564.

Supplementary methods. Sensitivity analysis

Several sensitivity analyses were conducted to test the robustness of our results. For the analyses of transition pattern A, several sensitivity analyses were conducted: 1) using different time intervals (1 day, 0.5 year, 1 year, and 3 years) to calculate the entering date of the prior state for participants entering different states on the same date; 2) excluding participants who entered different states on the same date; 3) excluding the events occurring within the first two years of follow-up; 4) imputing the missing covariates with multiple imputation. The latter two sensitivity analyses were also conducted for the analyses of transition pattern B.

Table S1. Outline of JoGH's Guidelines for Reporting Analyses of Big Data Repositories Open to the Public (GRABDROP) items

**JoGH guideline item**	**Purpose**
1. Please list all papers published by each co-author in previous three years that were based on secondary analysis of a big data repository	1. Han M, Zhou J, Wan Z, Chen M, Yan M, Feng W, Wang G, Zhang J, Zhang L, Yan L, Shao F, Gu Y. Remnant cholesterol, high-sensitivity C-reactive protein, and risk of incident coronary heart disease among patients with chronic kidney disease based on UK biobank. *Nutr Metab (Lond)*. 2025;22(1):83.<br>2. Wan Z, Zhou J, Han M, Chen M, Shao F, Gu Y. Proteomic signatures of life's essential 8 and incident atrial fibrillation among individuals with chronic kidney disease. *Nutr Metab (Lond)*. 2026.
2. Please explain the key elements of your study design and the use of the available datasets that make your study an original scientific contribution	Based on a large-scale, prospective cohort including 392,902 participants with a median follow-up of 13.49 years, we systematically assessed the role of frailty in all disease transition stages from healthy to first cardiometabolic-kidney disease (FCMKD), subsequently to cardiometabolic-kidney multimorbidity (CMKM), and further to death. Multi-state models were adopted to evaluate hazard ratios and 95% confidence intervals. The findings highlight the significance of managing frailty to promote the primary and secondary prevention of CMKM under the CKM health framework.
3. Please list all publications that addressed similar research questions in the same dataset	Prior studies based on this dataset mainly focused on exploring the impact of frailty on the

and indicate where you cited them in your paper	incidence of single CMKDs using the fragmented analyses. Our study adopted the multi-state models to evaluate and compare the impact of frailty on the incidence and progression of cardiometabolic-kidney multimorbidity (CMKM) simultaneously. We have cited them in the second paragraph of the Introduction and the third paragraph of the Discussion.<br>1. Hanlon P, Nicholl BI, Jani BD, Lee D, McQueenie R, Mair FS. Frailty and pre-frailty in middle-aged and older adults and its association with multimorbidity and mortality: a prospective analysis of 493 737 UK Biobank participants. *Lancet Public Health*. 2018;3:e323–e332.<br>2. Sun Y, Li W, Zhou Y, Wang B, Tan X, Lu Y, et al. Long-term changes in frailty and incident type 2 diabetes: A prospective cohort study based on the UK Biobank. *Diabetes Obes Metab*. 2024;26:3352–3360.<br>3. Yang H, Li Z, Zhang Y, Chang Q, Jiang J, Liu Y, et al. Associations between frailty, genetic predisposition, and chronic kidney disease risk in middle-aged and older adults: A prospective cohort study. *Maturitas*. 2024;187:108059.
4. Please explain how you addressed multiple testing through an appropriately rigorous statistical threshold and indicate this in the methods section	For the primary results, we focused on the magnitudes of the effect estimates by reporting the hazard ratios and 95% confidence intervals. In addition, we considered a two-sided *P*-value <0.05 statistically significant.
5. Please declare to what extent have AI chatbots been used in developing your paper and to which parts of the paper did they contribute	No artificial Intelligence chatbots were used in developing this paper.

Table S2. Frailty criteria.

Components	Question/measurement	Criteria
Weight loss	Self-reported: "Compared with one year ago, has your weight changed?"	1= "Yes - lost weight";<br>0= "No - weight about the same" or "Yes - gained weight"
Exhaustion	Self-reported: "Over the past two weeks, how often have you felt tired or had little energy?"	1= "More than half the days" or "nearly every day";<br>0= "Several days" or "Not at all"
Low physical activity	Self-reported: "In the last 4 weeks did you spend any time doing the following? (You can select more than one answer)";<br>"How many times in the last 4 weeks did you do light DIY?"	1= "None of the above" or "Light DIY activity [eg, pruning, watering the lawn]" with a frequency of once per week or less;<br>0= "Walking for pleasure", or "Other exercises" or "Strenuous sports" or "Heavy DIY activity (eg, weeding, lawn mowing, carpentry and digging)" or "Light DIY activity [eg, pruning, watering the lawn]" with a frequency of more than once per week
Slow walking pace	Self-reported: "How would you describe your usual walking pace?"	1= "Slow pace";<br>0= "Steady average pace" or "Brisk pace"

Low grip strength	Measured: Jamar J00105 hydraulic hand dynamometer	**Men:**<br>If BMI ≤24.0 kg/m^2 & grip strength ≤29 kg<br>If BMI 24.1 to 26.0 kg/m^2 & grip strength ≤30 kg<br>If BMI 26.1 to 28.0 kg/m^2 & grip strength ≤30 kg<br>If BMI >28.0 kg/m^2 & grip strength ≤32 kg<br>**Women:**<br>If BMI ≤23.0 kg/m^2 & grip strength ≤17 kg<br>If BMI 23.1 to 26.0 kg/m^2 & grip strength ≤17.3 kg<br>If BMI 26.1 to 29.0 kg/m^2 & grip strength ≤18 kg<br>If BMI >29.0 kg/m^2 & grip strength ≤21 kg

BMI, body mass index

Table S3. Baseline characteristics of included and excluded participants.

Characters	Included participants (N= 392,902)	Excluded participants (N= 27,905)	*P* value
Age (years)	56.33±8.06	56.59±8.30	<0.001
Men (%)	170,886 (43.49)	11,812 (42.33)	<0.001
White (%)	374,538 (95.61)	23,707 (88.20)	<0.001
Townsend index	-1.47±2.99	-0.52±3.40	<0.001
High education (%)	194,207 (49.82)	8698 (37.88)	<0.001
Current smoker (%)	39,381 (10.05)	3553 (13.26)	<0.001
Drinking ≥3 times a week (%)	178,145 (45.37)	9520 (35.30)	<0.001
Healthy diet (%)	48,324 (12.30)	2720 (10.03)	<0.001
Family history of CMD (%)	226,478 (57.84)	15,474 (56.35)	<0.001
Obesity (%)	82,955 (21.11)	6277 (24.45)	<0.001
Hypertension (%)	193,293 (49.20)	14,077 (50.45)	<0.001
Lipid-lowering drugs use (%)	38,301 (9.80)	2519 (9.29)	0.006
Elevated LDL-C (mmol/L)	288,070 (77.76)	17,131 (77.09)	0.019

CMD, cardiometabolic disease; LDL-C, low-density lipoprotein cholesterol.

Table S4. Transition probabilities over years for transition pattern A according to frailty status.

	Years	Transition probability (%)		
		Non-frailty	Pre-frailty	Frailty
**FCMKD survivor**				
	1	0.160	0.393	1.726
	2	0.341	0.819	3.159
	5	1.023	2.358	6.898
	10	2.487	5.249	8.891
	15	4.176	7.746	6.476
**CMKM survivor**				
	1	0.001	0.009	0.157
	2	0.006	0.034	0.556
	5	0.034	0.192	2.341
	10	0.170	0.857	5.175
	15	0.485	2.099	5.144
**Dead without CMKD**				
	1	0.021	0.054	0.309
	2	0.060	0.151	0.856
	5	0.226	0.570	3.047
	10	0.625	1.55	7.303
	15	1.263	3.055	12.091
**Dead with FCMKD**				
	1	0.003	0.016	0.435
	2	0.007	0.042	1.033
	5	0.028	0.168	3.494
	10	0.105	0.600	9.075
	15	0.299	1.573	15.784
**Dead with CMKM**				

	1	<0.001	0.001	0.089
	2	<0.001	0.003	0.346
	5	0.002	0.024	2.005
	10	0.014	0.184	9.662
	15	0.064	0.778	22.297

FCMKD, first cardiometabolic-kidney disease; CMKM, cardiometabolic-kidney multimorbidity

Table S5. Hazard ratios (95% CIs) for transition pattern A associated with frailty stratified by sex.

	Pre-frailty		Frailty		Per 1-indicator
	Cases	HR (95% CI)	Cases	HR (95% CI)	
**Men**					
Baseline → FCMKD	11456	1.27 (1.24-1.31)	963	1.89 (1.76-2.03)	1.23 (1.21-1.25)
FCMKD → CMKM	1940	1.12 (1.05-1.19)	241	1.55 (1.34-1.80)	1.15 (1.11-1.19)
Baseline → Death	3154	1.32 (1.26-1.38)	375	2.67 (2.37-3.01)	1.31 (1.28-1.35)
FCMKD → Death	1824	1.16 (1.08-1.24)	210	1.50 (1.28-1.76)	1.13 (1.09-1.17)
CMKM → Death	489	1.10 (0.97-1.26)	84	1.62 (1.25-2.09)	1.14 (1.06-1.21)
**Women**					
Baseline → FCMKD	11412	1.34 (1.31-1.38)	1739	2.20 (2.08-2.32)	1.27 (1.26-1.29)
FCMKD → CMKM	1739	1.18 (1.09-1.27)	348	1.43 (1.26-1.63)	1.13 (1.09-1.17)
Baseline → Death	3513	1.27 (1.21-1.34)	516	2.40 (2.17-2.66)	1.27 (1.24-1.30)
FCMKD → Death	1325	1.24 (1.14-1.35)	231	1.53 (1.31-1.79)	1.15 (1.10-1.20)
CMKM → Death	383	1.11 (0.94-1.32)	77	1.04 (0.79-1.37)	1.04 (0.97-1.12)

FCMKD, first cardiometabolic-kidney disease; CMKM, cardiometabolic-kidney multimorbidity; HR, hazard ratio.

Adjusted for age, ethnicity, region, education levels, Townsend deprivation index, smoking status, drinking status, healthy diet, family history of cardiometabolic disease, obesity, hypertension, lipid-lowering drugs use, and elevated low-density lipoprotein cholesterol.

Table S6. Hazard ratios (95% CIs) for transition pattern A associated with frailty stratified by age.

	Pre-frailty		Frailty		Per 1-indicator
	Cases	HR (95% CI)	Cases	HR (95% CI)	
**<60 years**					
Baseline → FCMKD	9672	1.35 (1.31-1.39)	1284	2.24 (2.10-2.39)	1.29 (1.27-1.31)
FCMKD → CMKM	1230	1.12 (1.02-1.22)	240	1.43 (1.23-1.67)	1.13 (1.08-1.18)
Baseline → Death	2339	1.31 (1.24-1.39)	357	2.73 (2.41-3.09)	1.31 (1.26-1.35)
FCMKD → Death	890	1.07 (0.97-1.18)	146	1.39 (1.15-1.69)	1.09 (1.04-1.15)
CMKM → Death	186	1.01 (0.80-1.27)	48	1.10 (0.76-1.59)	1.08 (0.98-1.19)
**≥60 years**					
Baseline → FCMKD	13,196	1.27 (1.24-1.31)	1418	1.92 (1.81-2.04)	1.23 (1.21-1.24)
FCMKD → CMKM	2449	1.15 (1.09-1.22)	349	1.47 (1.30-1.66)	1.13 (1.10-1.17)
Baseline → Death	4328	1.29 (1.24-1.35)	534	2.39 (2.17-2.64)	1.28 (1.25-1.31)
FCMKD → Death	2259	1.23 (1.16-1.31)	295	1.54 (1.34-1.76)	1.15 (1.12-1.19)
CMKM → Death	686	1.15 (1.02-1.29)	113	1.34 (1.07-1.67)	1.09 (1.03-1.16)

FCMKD, first cardiometabolic-kidney disease; CMKM, cardiometabolic-kidney multimorbidity; HR, hazard ratio.

Adjusted for sex, ethnicity, region, education levels, Townsend deprivation index, smoking status, drinking status, healthy diet, family history of cardiometabolic disease, obesity, hypertension, lipid-lowering drugs use, and elevated low-density lipoprotein cholesterol.

Table S7. Hazard ratios (95% CIs) for transition pattern B associated with frailty stratified by sex.

	Pre-frailty		Frailty		Per 1-indicator
	Cases	HR (95% CI)	Cases	HR (95% CI)	
**Men**					
Baseline → FCMKD					
Baseline → Diabetes	2669	1.45 (1.37-1.53)	272	2.20 (1.92-2.52)	1.32 (1.28-1.36)
Baseline → CKD	1403	1.22 (1.14-1.31)	106	1.76 (1.42-2.18)	1.20 (1.14-1.25)
Baseline → Stroke	1157	1.17 (1.08-1.26)	87	1.58 (1.24-2.02)	1.15 (1.10-1.21)
Baseline → CHD	5834	1.24 (1.20-1.28)	459	1.83 (1.65-2.03)	1.20 (1.18-1.23)
FCMKD → CMKM					
Diabetes → CMKM	387	0.98 (0.84-1.14)	65	1.53 (1.15-2.05)	1.11 (1.03-1.20)
CKD → CMKM	242	1.31 (1.09-1.57)	28	2.13 (1.39-3.26)	1.27 (1.16-1.41)
Stroke → CMKM	170	1.04 (0.84-1.28)	19	1.37 (0.79-2.37)	1.11 (0.98-1.26)
CHD → CMKM	748	1.13 (1.02-1.26)	90	1.63 (1.28-2.06)	1.17 (1.10-1.24)
Baseline → Death	3154	1.32 (1.26-1.39)	375	2.68 (2.38-3.03)	1.32 (1.28-1.35)
FCMKD → Death					
Diabetes → Death	258	1.03 (0.86-1.24)	37	1.47 (1.00-2.18)	1.09 (0.99-1.21)
CKD → Death	218	1.11 (0.92-1.33)	24	1.77 (1.12-2.78)	1.11 (1.00-1.24)
Stroke → Death	284	1.04 (0.88-1.22)	25	1.10 (0.67-1.78)	1.02 (0.92-1.13)
CHD → Death	1064	1.26 (1.16-1.38)	124	1.63 (1.32-2.01)	1.18 (1.13-1.24)
CMKM → Death	416	1.18 (1.02-1.36)	73	1.60 (1.22-2.11)	1.15 (1.08-1.24)
**Women**					
Baseline → FCMKD					
Baseline → Diabetes	2886	1.49 (1.40-1.59)	540	2.34 (2.10-2.60)	1.31 (1.27-1.35)
Baseline → CKD	2540	1.21 (1.14-1.28)	374	2.06 (1.83-2.32)	1.24 (1.20-1.27)
Baseline → Stroke	1250	1.26 (1.16-1.36)	169	2.21 (1.86-2.63)	1.25 (1.19-1.30)
Baseline → CHD	4401	1.39 (1.33-1.45)	594	2.20 (2.00-2.41)	1.28 (1.25-1.31)
FCMKD → CMKM					

Diabetes → CMKM	362	1.28 (1.06-1.55)	85	1.38 (1.03-1.84)	1.13 (1.04-1.22)
CKD → CMKM	292	1.32 (1.09-1.58)	71	2.14 (1.59-2.88)	1.28 (1.18-1.39)
Stroke → CMKM	163	1.40 (1.09-1.79)	22	1.37 (0.85-2.20)	1.12 (1.00-1.26)
CHD → CMKM	587	1.12 (0.98-1.27)	108	1.44 (1.15-1.80)	1.11 (1.04-1.18)
Baseline → Death	3513	1.27 (1.21-1.34)	516	2.41 (2.18-2.67)	1.27 (1.24-1.31)
FCMKD → Death					
Diabetes → Death	205	1.01 (0.81-1.26)	48	1.41 (0.99-2.01)	1.11 (1.00-1.22)
CKD → Death	256	1.37 (1.13-1.66)	43	1.67 (1.17-2.40)	1.23 (1.13-1.35)
Stroke → Death	351	1.35 (1.15-1.58)	47	1.17 (0.82-1.66)	1.12 (1.03-1.21)
CHD → Death	513	1.27 (1.10-1.46)	93	1.79 (1.41-2.29)	1.18 (1.10-1.26)
CMKM → Death	325	1.14 (0.95-1.37)	64	1.02 (0.76-1.39)	1.05 (0.97-1.14)

FCMKD, first cardiometabolic-kidney disease; CKD, chronic kidney disease; CHD, coronary heart disease; CMKM, cardiometabolic-kidney multimorbidity; HR, hazard ratio.

Adjusted for age, ethnicity, region, education levels, Townsend deprivation index, smoking status, drinking status, healthy diet, family history of cardiometabolic disease, obesity, hypertension, lipid-lowering drugs use, and elevated low-density lipoprotein cholesterol.

Table S8. Hazard ratios (95% CIs) for transition pattern B associated with frailty stratified by age.

	Pre-frailty		Frailty		Per 1-indicator
	Cases	HR (95% CI)	Cases	HR (95% CI)	
**<60 years**					
Baseline → FCMKD					
Baseline → Diabetes	2966	1.53 (1.45-1.62)	493	2.49 (2.23-2.78)	1.34 (1.31-1.38)
Baseline → CKD	1301	1.32 (1.22-1.43)	181	2.39 (2.01-2.83)	1.31 (1.25-1.37)
Baseline → Stroke	858	1.18 (1.07-1.29)	93	2.03 (1.63-2.54)	1.20 (1.14-1.27)
Baseline → CHD	4262	1.29 (1.24-1.35)	472	2.07 (1.87-2.30)	1.25 (1.22-1.28)
FCMKD → CMKM					
Diabetes → CMKM	290	0.99 (0.82-1.19)	75	1.35 (1.00-1.82)	1.10 (1.01-1.19)
CKD → CMKM	137	1.15 (0.89-1.49)	35	2.21 (1.46-3.35)	1.33 (1.19-1.49)
Stroke → CMKM	96	1.59 (1.17-2.17)	15	2.27 (1.23-4.19)	1.31 (1.13-1.53)
CHD → CMKM	422	1.14 (0.98-1.33)	70	1.39 (1.05-1.85)	1.12 (1.04-1.20)
Baseline → Death	2339	1.31 (1.24-1.39)	357	2.73 (2.41-3.09)	1.31 (1.27-1.35)
FCMKD → Death					
Diabetes → Death	149	0.95 (0.73-1.22)	46	1.89 (1.29-2.77)	1.16 (1.04-1.30)
CKD → Death	98	1.19 (0.88-1.60)	11	1.30 (0.68-2.51)	1.11 (0.95-1.30)
Stroke → Death	169	1.24 (1.00-1.54)	19	1.16 (0.67-2.02)	1.11 (0.98-1.25)
CHD → Death	474	1.10 (0.96-1.25)	70	1.37 (1.05-1.81)	1.10 (1.03-1.18)
CMKM → Death	159	1.16 (0.90-1.49)	43	1.20 (0.81-1.79)	1.12 (1.01-1.25)
**≥60 years**					
Baseline → FCMKD					
Baseline → Diabetes	2589	1.41 (1.32-1.49)	319	2.03 (1.78-2.31)	1.28 (1.24-1.32)
Baseline → CKD	2642	1.17 (1.11-1.24)	299	1.81 (1.59-2.06)	1.18 (1.15-1.22)
Baseline → Stroke	1549	1.22 (1.14-1.31)	163	1.88 (1.58-2.25)	1.20 (1.15-1.25)
Baseline → CHD	5973	1.29 (1.24-1.34)	581	1.94 (1.77-2.12)	1.23 (1.20-1.25)
FCMKD → CMKM					

Diabetes → CMKM	459	1.15 (0.99-1.33)	75	1.39 (1.05-1.84)	1.13 (1.05-1.22)
CKD → CMKM	397	1.37 (1.18-1.59)	64	1.99 (1.47-2.68)	1.25 (1.16-1.34)
Stroke → CMKM	237	1.06 (0.88-1.27)	26	1.03 (0.65-1.62)	1.04 (0.94-1.16)
CHD → CMKM	913	1.12 (1.02-1.23)	128	1.59 (1.30-1.94)	1.15 (1.09-1.21)
Baseline → Death	4328	1.29 (1.24-1.35)	534	2.40 (2.18-2.65)	1.28 (1.25-1.31)
FCMKD → Death					
Diabetes → Death	314	1.06 (0.89-1.26)	39	1.09 (0.75-1.59)	1.05 (0.96-1.15)
CKD → Death	376	1.23 (1.06-1.42)	56	1.72 (1.26-2.34)	1.19 (1.10-1.29)
Stroke → Death	466	1.17 (1.03-1.34)	53	1.14 (0.82-1.58)	1.08 (1.00-1.16)
CHD → Death	1103	1.34 (1.23-1.47)	147	1.88 (1.55-2.27)	1.22 (1.17-1.28)
CMKM → Death	582	1.17 (1.03-1.33)	94	1.31 (1.03-1.66)	1.10 (1.03-1.17)

FCMKD, first cardiometabolic-kidney disease; CKD, chronic kidney disease; CHD, coronary heart disease; CMKM, cardiometabolic-kidney multimorbidity; HR, hazard ratio.

Adjusted for sex, ethnicity, region, education levels, Townsend deprivation index, smoking status, drinking status, healthy diet, family history of cardiometabolic disease, obesity, hypertension, lipid-lowering drugs use, and elevated low-density lipoprotein cholesterol.

Table S9. Sensitivity analyses for transition pattern A.

	Pre-frailty	Frailty	Per 1-indicator
**Using 1 day as the time interval**			
Baseline → FCMKD	1.30 (1.28-1.33)	2.08 (1.99-2.17)	1.25 (1.24-1.27)
FCMKD → CMKM	1.14 (1.09-1.20)	1.47 (1.34-1.62)	1.13 (1.11-1.16)
Baseline → Death	1.30 (1.25-1.34)	2.52 (2.34-2.72)	1.29 (1.27-1.32)
FCMKD → Death	1.19 (1.13-1.25)	1.49 (1.33-1.67)	1.13 (1.10-1.17)
CMKM → Death	1.11 (1.00-1.23)	1.28 (1.06-1.54)	1.09 (1.04-1.14)
**Using 0.5 year as the time interval**			
Baseline → FCMKD	1.30 (1.28-1.33)	2.08 (1.99-2.17)	1.25 (1.24-1.27)
FCMKD → CMKM	1.14 (1.09-1.20)	1.47 (1.33-1.62)	1.13 (1.11-1.16)
Baseline → Death	1.30 (1.25-1.34)	2.52 (2.34-2.73)	1.29 (1.27-1.32)
FCMKD → Death	1.19 (1.13-1.25)	1.49 (1.34-1.67)	1.13 (1.10-1.17)
CMKM → Death	1.11 (1.00-1.23)	1.28 (1.06-1.54)	1.09 (1.04-1.15)
**Using 1 year as the time interval**			
Baseline → FCMKD	1.30 (1.28-1.33)	2.08 (1.99-2.17)	1.25 (1.24-1.27)
FCMKD → CMKM	1.14 (1.09-1.20)	1.47 (1.33-1.62)	1.13 (1.11-1.16)
Baseline → Death	1.30 (1.25-1.34)	2.52 (2.34-2.72)	1.29 (1.27-1.32)
FCMKD → Death	1.19 (1.13-1.25)	1.50 (1.34-1.68)	1.14 (1.10-1.17)
CMKM → Death	1.11 (1.00-1.23)	1.27 (1.05-1.53)	1.09 (1.04-1.14)
**Using 3 years as the time interval**			
Baseline → FCMKD	1.30 (1.28-1.33)	2.08 (1.99-2.17)	1.25 (1.24-1.27)
FCMKD → CMKM	1.14 (1.09-1.20)	1.46 (1.33-1.61)	1.13 (1.10-1.16)
Baseline → Death	1.30 (1.26-1.35)	2.52 (2.33-2.72)	1.29 (1.27-1.32)
FCMKD → Death	1.18 (1.12-1.24)	1.50 (1.34-1.68)	1.13 (1.10-1.17)
CMKM → Death	1.10 (1.00-1.23)	1.29 (1.06-1.55)	1.09 (1.04-1.14)
**Excluding participants who entered different states on the same date**			
Baseline → FCMKD	1.30 (1.28-1.33)	2.08 (1.99-2.18)	1.25 (1.24-1.27)
FCMKD → CMKM	1.16 (1.10-1.23)	1.56 (1.41-1.74)	1.16 (1.13-1.19)

Baseline → Death	1.30 (1.26-1.34)	2.53 (2.35-2.74)	1.29 (1.27-1.32)
FCMKD → Death	1.19 (1.13-1.25)	1.49 (1.33-1.67)	1.13 (1.10-1.17)
CMKM → Death	1.16 (1.03-1.30)	1.28 (1.04-1.57)	1.10 (1.05-1.16)
**Excluding the events occurring within the first two years of follow-up**			
Baseline → FCMKD	1.31 (1.28-1.33)	2.09 (2.00-2.19)	1.26 (1.24-1.27)
FCMKD → CMKM	1.14 (1.08-1.20)	1.36 (1.23-1.52)	1.12 (1.09-1.15)
Baseline → Death	1.28 (1.23-1.32)	2.39 (2.20-2.59)	1.27 (1.25-1.30)
FCMKD → Death	1.15 (1.09-1.21)	1.46 (1.30-1.64)	1.12 (1.09-1.15)
CMKM → Death	1.11 (0.99-1.24)	1.21 (0.98-1.49)	1.08 (1.02-1.14)
**Adjusting for the imputed covariates with multiple imputation**			
Baseline → FCMKD	1.30 (1.28-1.33)	2.03 (1.95-2.12)	1.25 (1.24-1.26)
FCMKD → CMKM	1.15 (1.10-1.21)	1.47 (1.34-1.61)	1.13 (1.11-1.16)
Baseline → Death	1.30 (1.26-1.34)	2.56 (2.38-2.74)	1.30 (1.28-1.32)
FCMKD → Death	1.20 (1.14-1.26)	1.54 (1.39-1.71)	1.15 (1.12-1.18)
CMKM → Death	1.10 (1.00-1.21)	1.26 (1.05-1.50)	1.08 (1.03-1.13)

FCMKD, first cardiometabolic-kidney disease; CMKM, cardiometabolic-kidney multimorbidity. Adjusted for age, sex, ethnicity, region, education levels, Townsend deprivation index, smoking status, drinking status, healthy diet, family history of cardiometabolic disease, obesity, hypertension, lipid-lowering drugs use, and elevated low-density lipoprotein cholesterol.

Table S10. Sensitivity analyses for transition pattern B.

	Pre-frailty	Frailty	Per 1-indicator
**Excluding the events occurring within the first two years of follow-up**			
Baseline → FCMKD			
Baseline → Diabetes	1.47 (1.41-1.53)	2.27 (2.08-2.47)	1.31 (1.28-1.34)
Baseline → CKD	1.25 (1.19-1.31)	2.13 (1.92-2.38)	1.25 (1.21-1.28)
Baseline → Stroke	1.20 (1.13-1.27)	2.02 (1.75-2.33)	1.21 (1.17-1.25)
Baseline → CHD	1.29 (1.25-1.32)	1.96 (1.82-2.11)	1.23 (1.21-1.25)
FCMKD → CMKM			
Diabetes → CMKM	1.12 (0.99-1.26)	1.34 (1.08-1.66)	1.11 (1.05-1.18)
CKD → CMKM	1.27 (1.10-1.46)	1.84 (1.41-2.41)	1.24 (1.16-1.33)
Stroke → CMKM	1.11 (0.93-1.32)	1.18 (0.79-1.75)	1.08 (0.98-1.19)
CHD → CMKM	1.14 (1.04-1.24)	1.48 (1.23-1.78)	1.12 (1.07-1.17)
Baseline → Death	1.28 (1.23-1.32)	2.40 (2.21-2.60)	1.27 (1.25-1.30)
FCMKD → Death			
Diabetes → Death	1.01 (0.87-1.17)	1.49 (1.15-1.94)	1.10 (1.03-1.18)
CKD → Death	1.14 (0.99-1.31)	1.49 (1.12-2.00)	1.14 (1.06-1.22)
Stroke → Death	1.18 (1.06-1.33)	1.04 (0.78-1.40)	1.06 (0.99-1.13)
CHD → Death	1.23 (1.13-1.33)	1.70 (1.44-2.01)	1.17 (1.13-1.22)
CMKM → Death	1.16 (1.03-1.31)	1.17 (0.93-1.47)	1.09 (1.02-1.15)
**Adjusting for the imputed covariates with multiple imputation**			
Baseline → FCMKD			
Baseline → Diabetes	1.48 (1.42-1.54)	2.24 (2.07-2.42)	1.31 (1.28-1.33)
Baseline → CKD	1.22 (1.17-1.27)	1.98 (1.80-2.18)	1.22 (1.19-1.25)
Baseline → Stroke	1.20 (1.14-1.26)	1.92 (1.69-2.19)	1.20 (1.16-1.23)
Baseline → CHD	1.29 (1.26-1.33)	1.95 (1.82-2.08)	1.24 (1.22-1.25)
FCMKD → CMKM			
Diabetes → CMKM	1.09 (0.97-1.21)	1.46 (1.21-1.76)	1.13 (1.07-1.18)
CKD → CMKM	1.31 (1.16-1.49)	2.17 (1.73-2.72)	1.28 (1.20-1.35)

Stroke → CMKM	1.20 (1.03-1.39)	1.31 (0.94-1.84)	1.11 (1.02-1.20)
CHD → CMKM	1.14 (1.06-1.23)	1.45 (1.24-1.69)	1.13 (1.09-1.18)
Baseline → Death	1.30 (1.26-1.34)	2.56 (2.39-2.75)	1.30 (1.28-1.32)
FCMKD → Death			
Diabetes → Death	1.02 (0.89-1.17)	1.45 (1.14-1.85)	1.10 (1.03-1.18)
CKD → Death	1.21 (1.07-1.37)	1.64 (1.26-2.13)	1.17 (1.09-1.25)
Stroke → Death	1.17 (1.05-1.30)	1.32 (1.03-1.69)	1.10 (1.04-1.17)
CHD → Death	1.30 (1.21-1.39)	1.70 (1.46-1.97)	1.19 (1.15-1.24)
CMKM → Death	1.14 (1.02-1.26)	1.24 (1.02-1.50)	1.09 (1.04-1.15)

FCMKD, first cardiometabolic-kidney disease; CKD, chronic kidney disease; CHD, coronary heart disease; CMKM, cardiometabolic-kidney multimorbidity.

Adjusted for age, sex, ethnicity, region, education levels, Townsend deprivation index, smoking status, drinking status, healthy diet, family history of cardiometabolic disease, obesity, hypertension, lipid-lowering drugs use, and elevated low-density lipoprotein cholesterol.

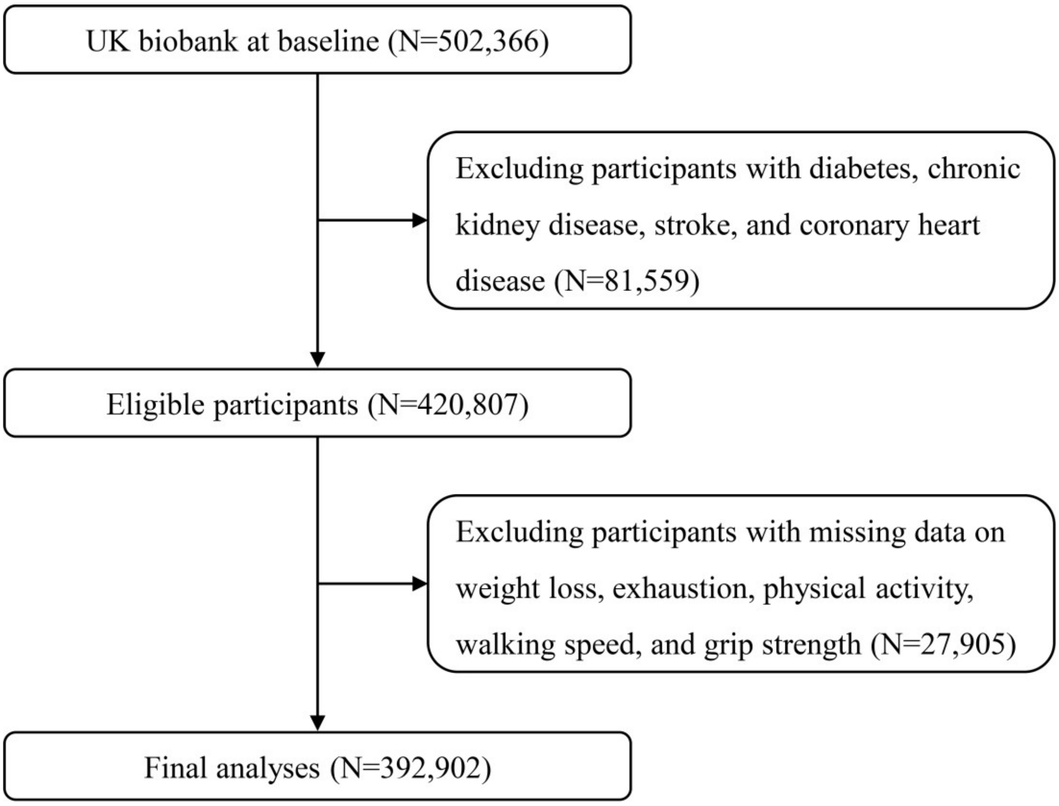

Figure S1. Flow chart of study participants included and excluded in the analyses.

STROBE Statement—Checklist of items that should be included in reports of ***cohort studies***

	Item No	Recommendation	Pages
**Title and abstract**	1	(*a*) Indicate the study's design with a commonly used term in the title or the abstract	Page 1 and Page 3
		(*b*) Provide in the abstract an informative and balanced summary of what was done and what was found	Page 3
**Introduction**			
Background/rationale	2	Explain the scientific background and rationale for the investigation being reported	Page 4
Objectives	3	State specific objectives, including any prespecified hypotheses	Pages 4-5
**Methods**			
Study design	4	Present key elements of study design early in the paper	Page 5
Setting	5	Describe the setting, locations, and relevant dates, including periods of recruitment, exposure, follow-up, and data collection	Pages 5-7
Participants	6	(*a*) Give the eligibility criteria, and the sources and methods of selection of participants. Describe methods of follow-up	Page 5
		(*b*) For matched studies, give matching criteria and number of exposed and unexposed	NA
Variables	7	Clearly define all outcomes, exposures, predictors, potential confounders, and effect modifiers. Give diagnostic criteria, if applicable	Pages 5-7
Data sources/ measurement	8*	For each variable of interest, give sources of data and details of methods of assessment (measurement). Describe comparability of assessment methods if there is more than one group	Pages 5-7
Bias	9	Describe any efforts to address potential sources of bias	Pages 7-8
Study size	10	Explain how the study size was arrived at	Page 5
Quantitative variables	11	Explain how quantitative variables were handled in the analyses. If applicable, describe which groupings were chosen and why	Pages 5-7
Statistical methods	12	(*a*) Describe all statistical methods, including those used to control for confounding	Pages 7-8
		(*b*) Describe any methods used to examine subgroups and interactions	Page 8
		(*c*) Explain how missing data were addressed	Page 8
		(*d*) If applicable, explain how loss to follow-up was addressed	Page 5
		(*e*) Describe any sensitivity analyses	Page 8
**Results**			
Participants	13*	(a) Report numbers of individuals at each stage of study—eg	Page 5 and

		numbers potentially eligible, examined for eligibility, confirmed eligible, included in the study, completing follow-up, and analysed	page 8
		(b) Give reasons for non-participation at each stage	Page 5
		(c) Consider use of a flow diagram	Page 5
Descriptive data	14*	(a) Give characteristics of study participants (eg demographic, clinical, social) and information on exposures and potential confounders	Page 8
		(b) Indicate number of participants with missing data for each variable of interest	Page 5
		(c) Summarise follow-up time (eg, average and total amount)	Page 8
Outcome data	15*	Report numbers of outcome events or summary measures over time	Page 8
Main results	16	(*a*) Give unadjusted estimates and, if applicable, confounder-adjusted estimates and their precision (eg, 95% confidence interval). Make clear which confounders were adjusted for and why they were included	Pages 9-11
		(*b*) Report category boundaries when continuous variables were categorized	Pages 9-10
		(*c*) If relevant, consider translating estimates of relative risk into absolute risk for a meaningful time period	NA
Other analyses	17	Report other analyses done—eg analyses of subgroups and interactions, and sensitivity analyses	Pages 10-11
**Discussion**			
Key results	18	Summarise key results with reference to study objectives	Page 11
Limitations	19	Discuss limitations of the study, taking into account sources of potential bias or imprecision. Discuss both direction and magnitude of any potential bias	Pages 13-14
Interpretation	20	Give a cautious overall interpretation of results considering objectives, limitations, multiplicity of analyses, results from similar studies, and other relevant evidence	Pages 11-14
Generalisability	21	Discuss the generalisability (external validity) of the study results	Pages 11-14
**Other information**			
Funding	22	Give the source of funding and the role of the funders for the present study and, if applicable, for the original study on which the present article is based	Page 15

*Give information separately for exposed and unexposed groups.

Note: An Explanation and Elaboration article discusses each checklist item and gives methodological background and published examples of transparent reporting. The STROBE checklist is best used in conjunction with this article (freely available on the Web sites of PLoS Medicine at http://www.plosmedicine.org/, Annals of Internal Medicine at http://www.annals.org/, and

Epidemiology at http://www.epidem.com/). Information on the STROBE Initiative is available at http://www.strobe-statement.org.
